# Habitat characteristics and climatic factors influence microhabitat selection and arthropod community structure in a globally rare central Appalachian shale barren

**DOI:** 10.1002/ece3.8413

**Published:** 2021-12-01

**Authors:** Andrew P. Landsman, Clara R. Thiel

**Affiliations:** ^1^ National Park Service United States Department of the Interior Williamsport Maryland USA

**Keywords:** arthropod community, epigeic insects, microhabitat, shale barrens, thermoregulation

## Abstract

1. The central Appalachian shale barrens, a globally unique habitat type restricted to the eastern United States, presents an insular and physiologically stressful environment with sparse vegetation and extreme ground surface and air temperatures. Despite the high levels of plant species endemism within these systems, information on invertebrate communities and habitat preferences is extremely limited.
2. Through this study, we aimed to better understand a shale barren arthropod community, microhabitat selection, and the influence of habitat characteristics and climatic factors. We employed pitfall traps to sample epigeic arthropods during the 2016 growing season in a shale barren habitat.
3. Arthropod community composition was driven by overstory trees, mediated through accumulated leaf litter and availability of shaded microhabitats. Ambient air temperature also influenced the surface activity of various taxa with spiders decreasing at higher temperatures and ants, crickets, flies, and harvestmen all increasing in relative abundance.
4. Habitat integrity of the central Appalachian shale barrens is threatened by forest succession and mesophication, encroaching invasive plant species, and rising ambient air temperatures, all of which can alter the extent of overstory vegetation and availability of shaded microhabitats. These biotic and physical pressures will subsequently affect epigeic arthropod community composition, depending on adaptive capacity of individual taxa.
5. To the authors’ knowledge, these findings constitute only the second published work on arthropod communities and the first to focus on epigeic taxa in this globally rare habitat type. Continued conservation of these unique, insular habitats and their adapted inhabitants requires a multifaceted approach that considers current and future conditions.

## Graphical abstract

Globally rare central Appalachian shale barrens exhibit high levels of plant endemism, yet extremely little is known about the wildlife communities therein. We uncovered the importance of overstory trees, mediated by shaded microhabitats and accumulated leaf litter, in predicting epigeic arthropod community composition. Given these results, current and future conservation threats to these unique habitats may differentially affect various invertebrate taxa, depending on adaptive capacity and resiliency to environmental change.

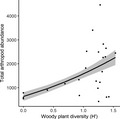

## 1 INTRODUCTION

Understanding microhabitat use and selection by terrestrial animals is critical to the conservation and management of plants and wildlife, especially threatened and unique species and communities. Local, fine‐scale habitat heterogeneity allows for the spatial distribution of various microhabitats throughout a single, dominant habitat type. This heterogeneity provides differing resources that facilitate the nonrandom distribution of animals across the landscape and may help to avoid realized niche overlap (Bates et al., 2007; Ramey & Richardson, 2017). Microhabitat selection and use may be driven by individual species’ life history requirements, environmental factors (e.g., plant cover and shade), biological pressures (e.g., competition), and ability to withstand climatic stressors. For terrestrial arthropods, selection of microhabitats often depends on a range of factors, including habitat structure (Landsman & Bowman, 2017; Miyashita & Takada, 2007), availability and evidence of available prey (De Omena & Romero, 2008; Johnson et al., 2011; Landsman et al., 2020; Morais‐Filho & Romero, 2008), quality and quantity of floral resources (Ruttan et al., 2016), nesting material (Beiroz et al., 2016), sites for oviposition (Bergman, 1999), competition (Wittman et al., 2010), and survival rates for larvae (Albanese et al., 2008). Another component of habitat selection, perhaps the most critical for some invertebrate groups, is the functional separation of microhabitats based on fine‐scale microclimates (May, 1979; Stevenson, 1985).

Many invertebrate taxa actively select favorable microclimates to avoid desiccation or mortality, facilitate organismal development, and to optimize foraging and metabolic efficiencies (Stevenson, 1985). Other, less vagile taxa may experience fluctuations in development rates and mortality risks due to the inability to move between microhabitats that differ in ambient air temperature or solar irradiation (Pincebourde et al., 2007). While much of the literature on the relationship between climate and insect oviposition has focused on Lepidoptera (Albanese et al., 2008; Bergman, 1999; Thom & Daniels, 2017), thermal constraints imposed by air and ground surface temperatures are similarly important for epigeic taxa, with temperatures driving community composition, selection of oviposition sites, and surface activity of many species (Cornelisse & Hafernik, 2009; Warren et al., 2019; Wittman et al., 2010). Particularly in xeric habitats, the availability, extent, and distribution of shaded microhabitats allow for ectothermic organisms to effectively regulate body temperatures (Kearney et al., 2009; May, 1979). Many unique, high biodiversity habitats, including glades, barrens, and granitic outcrops, present physically and physiologically stressful environments often caused by such high temperatures (Cartwright, 2019). Despite the physical demands, these high stress habitats can provide a breadth of microhabitat diversity, ultimately facilitating an increased biological diversity across the landscape (Speziale & Ezcurra, 2014). These areas present unique systems within which to study arthropod microhabitat selection and how environmental factors interact with local climate to affect the distribution and community composition of various taxa.

As first described in the central Appalachian Mountains by Steele (1911), shale barrens are open, sparsely vegetated communities that occur on steep, south‐facing slopes comprised of thin layers of friable shale fragments (Keener, 1983). These habitats are disparately distributed throughout the Ridge and Valley province from central Pennsylvania to southwestern Virginia and adjacent West Virginia (Braunschweig et al., 1999; Wherry, 1930). Central Appalachian shale barrens are characterized by shallow, nutrient‐poor soils with limited organic matter and are overlain by dry, fissile fragments of shale and siltstone (Keener, 1983). In tandem with the soil profile, exposure to strong winds, high levels of solar irradiation, and rapid evaporation of surface moisture produces a sparse vegetation comprised predominantly of endemic species able to thrive under harsh solar, edaphic, and hydrologic conditions (Platt, 1951; Steele, 1911). Endemic shale barren plant species are obligate heliophytes and exhibit a low tolerance for competition with other plants (Platt, 1951). Despite this, the distribution and presence of shale barren endemic species is still driven edaphically, as the unique topography and soil profiles of shale barren environments provide suitable habitat for endemic species while proving too harsh for potential competitors (Keener, 1983; Platt, 1951). Shale barren habitats are relatively self‐sustaining due to the apparent and consistent stress regimes associated with their unique geological and topographical profiles (Braunschweig et al., 1999). Changes in land use, fire suppression, invasion of exotic species, and forest succession on habitat edges have contributed to shale barren habitat loss and degradation (Tyndall, 2015), which can subsequently alter habitat suitability for resident species.

The inherent physiological and environmental stressors faced by plants found in shale barrens similarly affect invertebrate inhabitants. Owing to the extreme temperatures and sparse vegetation, some arthropod fauna may reach or near physiological thresholds or critical thermal maxima. High temperatures, extensive solar irradiation, and conductive heating from rock surfaces can create physiologically stressful environments for poikilothermic organisms, particularly for epigeic taxa. While some physiological mechanisms exist for regulating body temperature, arthropod thermoregulatory behaviors are more important for ensuring body temperatures do not exceed critical limits (Stevenson, 1985). Selection of shaded microhabitats, either seasonally or diurnally, is the most commonly employed and effective means by which ectotherms regulate body temperatures in light of excessive ambient air temperatures (Humphreys, 1987; Karasov & Martínez del Rio, 2007; May, 1979). Shuttling between shaded areas in warmer air temperatures and sunny patches in cooler temperatures helps to optimize rates of physiological processes such as metabolism and to reduce mortality risks associated with overheating (Stevenson, 1985). In addition to selecting shaded microhabitats, certain arthropods utilize other behavioral mechanisms to regulate body temperature. Some taxa will alter diel activity patterns to avoid the warmest portions of the day with some species shifting from diurnal foraging patterns to completely nocturnal (Gamboa, 1976; Wittman et al., 2010). Social ants can move deeper into the soil in high temperatures, concurrently moving larvae to ensure continued development and survival (Jones & Oldroyd, 2006; Seeley & Heinrich, 1981). Other species, including cursorial beetles and Orthoptera, may also extend their legs to shift bodies further from the warmed ground surface (May, 1979).

Research in shale barren systems has largely focused on the unique communities of endemic plant species whereby little has been documented regarding arthropod communities or of microhabitat selection and use of these habitats. The scant work that has been done has focused on pollinators of endemic and other entomophilous plant species (Kalhorn et al., 2003), specific phytophagous families (Wheeler et al., 1983), or exotic and incidental captures (Wheeler, 1999, 2000). Kalhorn et al. (2003), in their study of bees among barren openings, edges, and forest, documented significantly more bees within open areas of sparse to no vegetation. In this study, our objectives were to (1) establish a better understanding of ground‐dwelling arthropod community dynamics in a globally rare Appalachian shale barren habitat, (2) determine the importance of local habitat traits and climatic conditions in predicting the relative abundance of various arthropod taxa, and (3) identify future and extant stressors that may disproportionately affect the conservation of these unique habitats. We anticipated to detect strong negative correlations between many taxa and covariates that influence ground surface temperatures, such as canopy cover, bare ground, and greater air temperatures. We also predicted a greater abundance of web‐building spiders and herbivorous insects with increased vegetation ground cover and species diversity. Understanding the influence of environmental covariates and the importance of microhabitat selection will facilitate more effective conservation of invertebrates within these unique habitats.

## 2 MATERIALS AND METHODS

### 2.1 Study area

We sampled arthropods in a central Appalachian shale barren comprised of Upper Devonian shale in the Foreknobs formation, approximately 365 to 460 million years old. The sampled shale barren consisted of both U.S. National Park Service and Maryland Department of Natural Resources properties near Little Orleans, Maryland. Based on the large proportion of bare rock ground cover and sparse vegetation, our study area was representative of an exemplary shale barren community and was located outside of the more vegetated habitat margins.

### 2.2 Sampling design

Within the study area, we placed 24 pitfall traps approximately along two elevation contours between 270 and 310 m above sea level (NAVD 88), with traps spaced approximately 30 m apart. We placed traps 30 m apart to ensure independence of individual traps: spacing of 30 m between traps was the greatest distance noted in the manuscripts analyzed in a recent meta‐analysis of pitfall trap studies of epigeic arthropods (Brown & Matthews, 2016) Traps each consisted of a 450‐mL plastic cup buried such that the rim of the cup was even with the ground surface and filled with a mixture of 50% propylene glycol, diluted with water. We used white plastic 3‐gal bucket lids, elevated approximately 12 cm above the cup, to prevent precipitation from entering the traps. We collected specimens and refilled trap liquid in approximately two‐week intervals, depending on extent of local precipitation. Traps were in operation from June through October 2016. We identified spiders to taxonomic family and other arthropods to order. We separated ants (Formicidae) from remaining Hymenoptera because of the vastly different life history. At each trap site, we placed a 1‐m^2^ plot centered around the trap and visually estimated percent ground cover, categorized by bare rock, vegetation, and leaf litter. As ground surface temperatures can vary greatly with shading, we used a spherical densitometer, held 1.2 m above the trap, to estimate percent canopy cover. Canopy and ground cover estimates were independently made by three individuals and the mean estimate was used in analyses. We also identified the presence of all vascular plant species within a 5‐m radius circular plot and identified all woody plant species and recorded the abundance of stems within a 15‐m radius plot, both centered on the trap. We calculated the Shannon diversity index of woody plant species using stem abundance at each trap site and Jaccard's diversity index for the presence and absence of all herbaceous and woody plants.

### 2.3 Data analysis

To understand relationships of arthropod species composition and community structure, we used package mvabund in R 4.0.3 to fit multivariate generalized linear models to our arthropod data (R Core Team, 2020; Wang et al., 2012, 2019). We separately analyzed the spider community data at the family level and the entire arthropod community at the taxonomic order. For multivariate models, we summed captures across the study period to calculate abundance of each group at each trap. Models were fit using ground cover and canopy cover estimates, woody plant diversity, and all plant diversity as covariates and we assessed model significance using 2000 bootstrapped iterations of PIT‐residuals (Warton et al., 2017; Westfall & Young, 1993). We then separately analyzed each insect order and spider family using univariate negative binomial generalized linear models and our ground cover, canopy cover, and vegetation indices. Owing to covariance between estimates of rock, vegetation, and leaf litter ground cover, these variables were analyzed in separate, individual models with each taxonomic group.

In addition to analyzing the arthropod community and the ground cover, vegetation, and canopy cover covariates, we also examined temporal patterns of arthropod groups for correlation with climatic variables. For each collection day, we noted the total precipitation and the mean daily maximum air temperature reached during the collection period. These climatic data were downloaded from the nearest weather station (USC00182282) included in the Global Historical Climatology Network, which we accessed through the National Oceanic and Atmospheric Administration's National Centers for Environmental Information (https://www.ncei.noaa.gov). Air temperature and precipitation during sample collection periods were included as covariates in linear mixed models with individual trap as a random effect in package nlme to predict the abundance of the various arthropod groups throughout the duration of the study period (Pinheiro et al., 2020). Finally, for illustrative purposes, we used the projected changes in average annual air temperature for this unit of the U.S. National Park Service (Chesapeake and Ohio Canal National Historical Park) as described by Gonzalez et al. (2018) to identify how warmer temperatures may affect shale barren arthropod communities. We used the fitted linear mixed effect models to predict individual taxa response to increased air temperatures. These statistically downscaled predictions were made using the ensemble means from general circulation models under representative concentration pathway (RCP) 8.5, which were used to compare temperatures from 1971 to 2000 and projected temperatures from 2071 to 2100. Under this RCP 8.5 scenario, annual average temperatures were expected to increase by 5.0 ± 1.0°C between the historical period and the projected period ending in 2100 (Gonzalez et al., 2018). Precipitation was expected to increase by 14.0 ± 7.0% by 2100.

## 3 RESULTS

**FIGURE 1:**
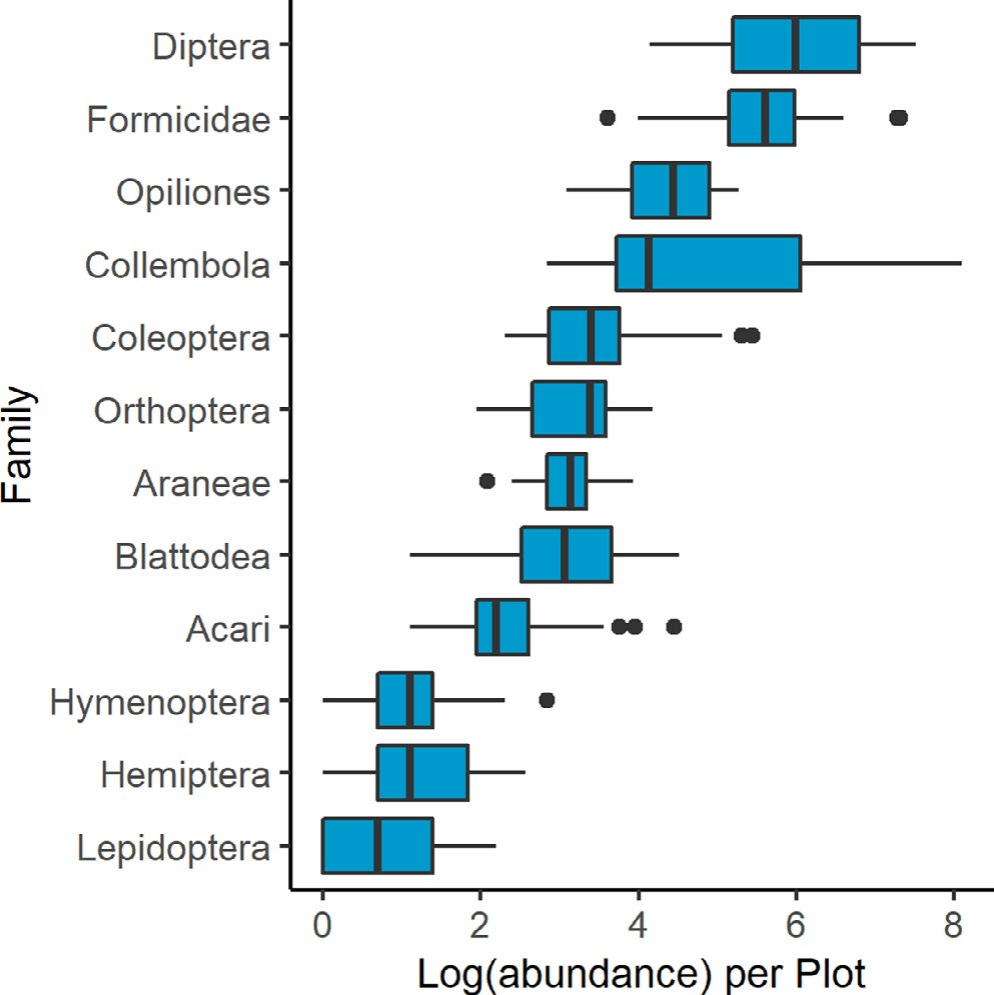
Boxplot showing logarithm of total abundance of 12 most abundant taxonomic orders

**FIGURE 2:**
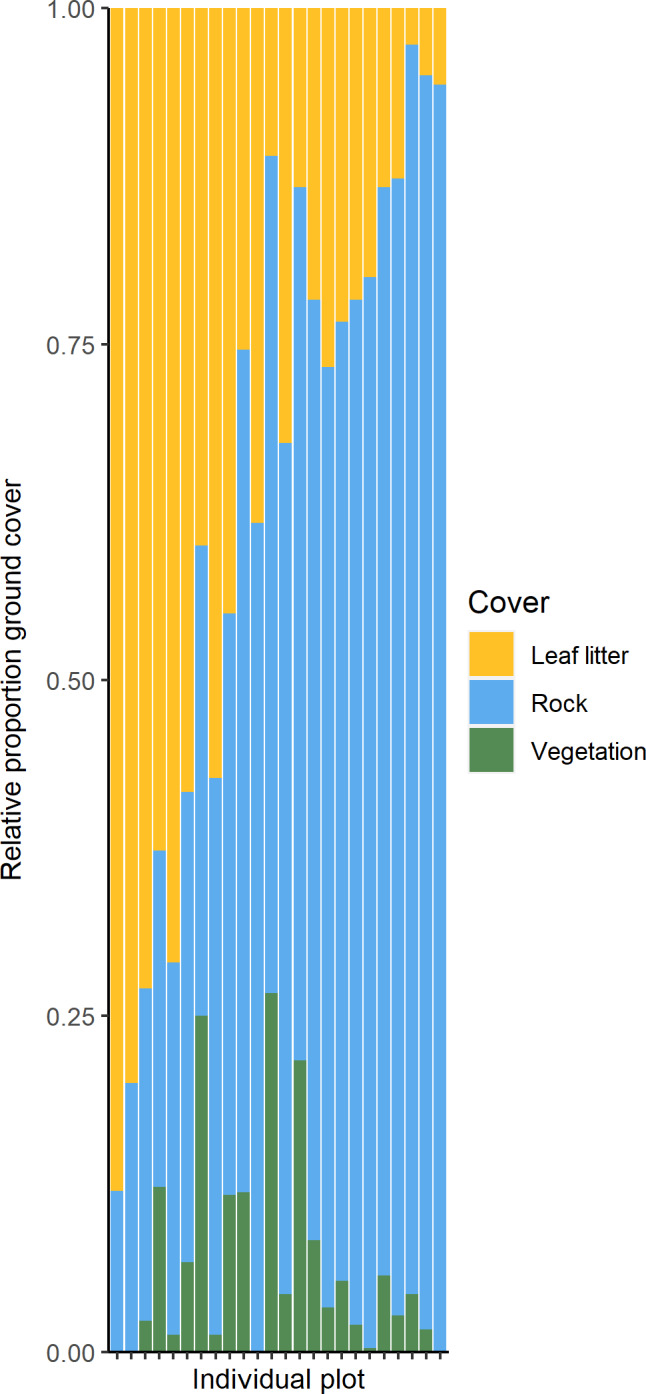
Stacked bar chart showing relative proportion of vegetation, rock, and leaf litter ground cover in 24 plots throughout study area

We collected a total of 40,638 arthropods from 24 distinct taxonomic orders (Figure 1). Of these specimens, we collected 1291 spiders but removed 728 recently hatched spiderlings before analysis. Flies (Diptera), springtails (Collembola), and ants (Hymenoptera: Formicidae) were the most abundant groups, comprising approximately 82% of total specimens, although we also collected numerous beetles (Coleoptera), crickets (Orthoptera: Gryllidae and Rhaphidophoridae), and harvestmen (Opiliones). Canopy cover estimates ranged from 5 to nearly 82%, with mean ± SE of 50.63 ± 3.98%. Within the 5‐m radius circle surrounding pitfall traps, we identified 43 plant species, including trees, vines, shrubs, grasses, ferns, and herbaceous plants. *Danthonia spicata*, a perennial grass, was the most commonly detected understory species, occurring in over 70% of plots. Species richness ranged from 2 to 18, with mean 7.75 individual plant species occurring in each plot. In 15‐m radius plots, we identified 360 individual plants comprising 15 woody shrub and tree species. Oak species (*Quercus montana*, *Q*. *rubra*, and *Q*. *ilicifolia*) dominated the overstory with over 56% of all trees. *Pinus virginiana*, *Juniperus virginiana*, and *Fraxinus* spp. were also commonly documented. We also noted six woody shrub species, including *Lonicera maackii*, an invasive exotic species. Vegetation ground cover estimates reached up to nearly 27% (Figure 2); however, most plots contained much less vegetation, with mean 6.61 ± 1.61%. Rock and bare soil comprised the most prevalent ground cover, with mean 57.97 ± 5.15%, and leaf litter cover represented mean 35.28 ± 5.23%. Daily mean maximum temperature for collection periods reached a high of 33.43°C with the highest daily air temperature documented at 37.78°C.

**FIGURE 3:**
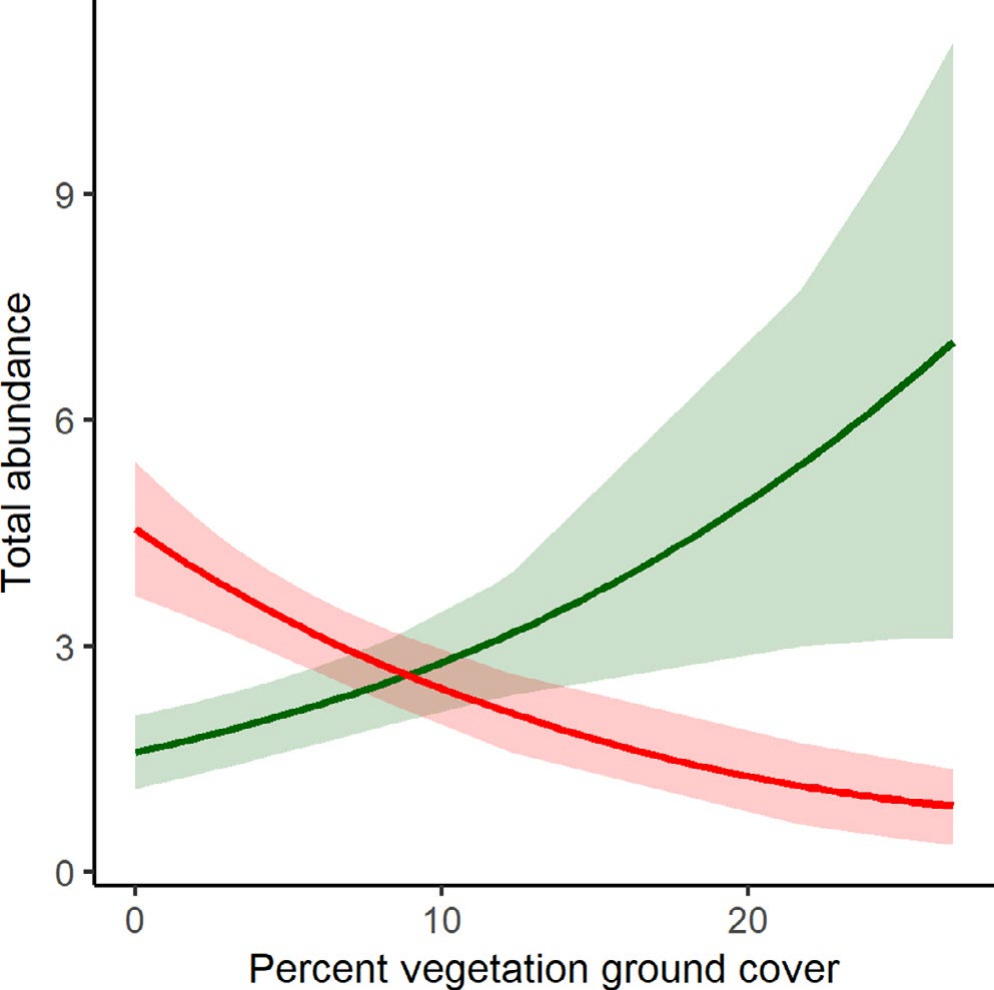
Total predicted abundance of the jumping spiders (Salticidae) and ground spiders (Gnaphosidae) across percent vegetation ground cover as estimated in a 1‐m^2^ square plot, centered on the pitfall trap. The Salticidae are represented by the green line, the Gnaphosidae are represented by the red line, and shading indicates predicted values ± SE

The most parsimonious multivariate model of spider families included both canopy cover (*G^2^* = 32.872; *p* = .021) and ground vegetation (*G^2^* = 26.979; *p* = .077). Local abundance of total individual spiders was not correlated with our environmental covariates. Family‐level analysis showed that only the jumping spiders in the Salticidae and the ground spiders in the Gnaphosidae were correlated with ground cover: jumping spiders increased with more vegetation cover (*z* = 2.069; *p* = .039) whereas ground‐dwelling Gnaphosidae exhibited an opposite, negative correlation with vegetation cover (*z* = −2.428; *p* = .015; Figure 3).

**FIGURE 4:**
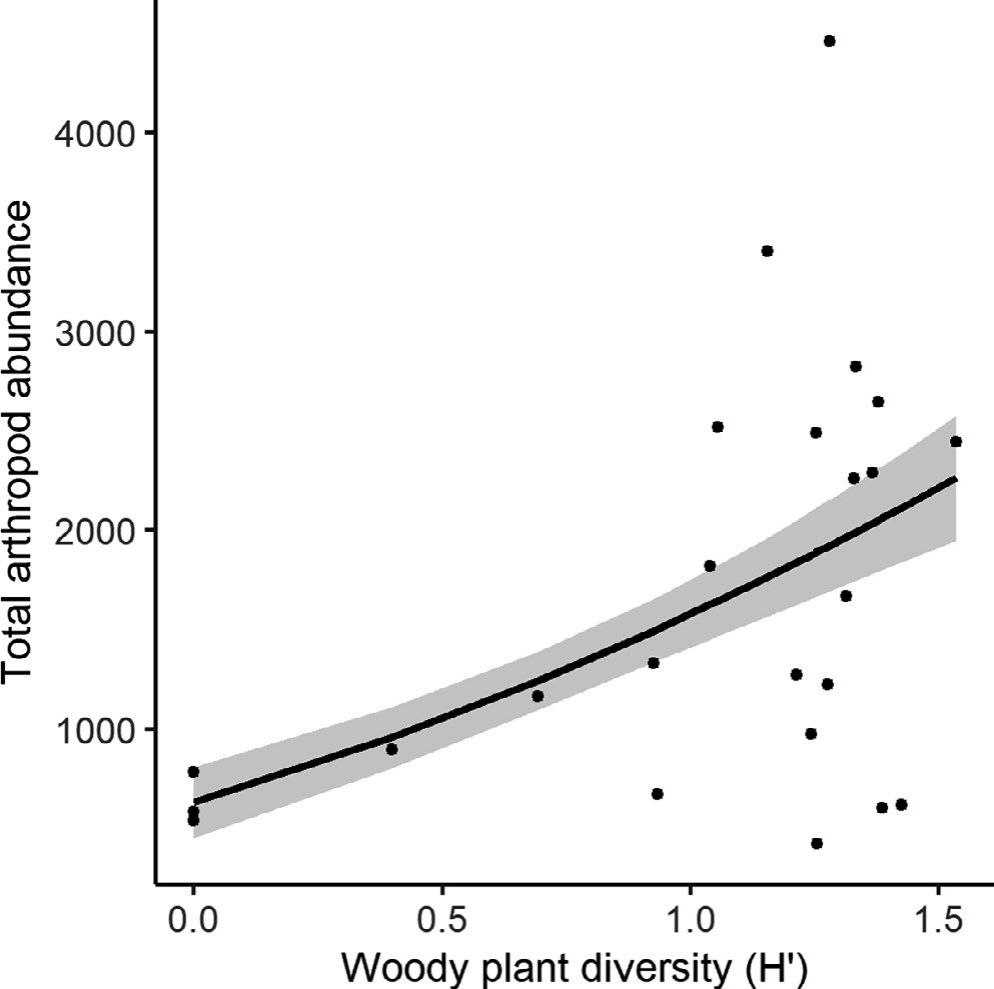
Relationship between total arthropod abundance and woody plant species diversity as calculated within a 15‐m radius circular plot surrounding the pitfall trap. Gray shading indicates predicted values ± SE

Considering all collected non‐spider arthropods, both the Shannon diversity of woody plants (*G^2^* = 48.228; *p* = .047) and the Jaccard diversity of all plant species (*G^2^* = 48.140; *p* = .037) were significant in predicting community structure. Similarly, woody plant diversity was positively correlated with the total number of arthropods (*z* = 4.096; *p *< .001; Figure 4). Individual taxonomic groups displayed significant correlations with various environmental covariates (Table 1). Canopy cover, woody plant diversity, and rock ground cover were correlated with more groups than was vegetation or leaf litter cover, and the abundance of many arthropod groups was best predicted using multiple covariates (Table 2).

**TABLE 1:** Correlation between abundance of arthropod taxa and environmental covariates surrounding pitfall traps collected in a central Appalachian shale barren in Maryland, U.S.A. in 2016

Taxon	Rock cover		Leaf Litter cover		Vegetation cover		Canopy cover		Woody plant diversity	
	*z*	*p*	*z*	*p*	*z*	*p*	*z*	*p*	*z*	*p*
Acari	1.322	n.s.	−0.787	n.s.	−**2.201**	.**0278**	−**2.157**	.**0310**	−**3.562**	.**0004**
Araneae	−0.913	n.s.	1.018	n.s.	−0.690	n.s.	0.975	n.s.	−1.109	n.s.
Blattodea	0.313	n.s.	−0.041	n.s.	−0.890	n.s.	**2.229**	.**0258**	**2.906**	.**0037**
Coleoptera	**2.658**	.**0079**	−**2.547**	.**0109**	−0.907	n.s.	−**2.619**	.**0088**	**3.136**	.**0017**
Collembola	−**2.971**	.**0030**	1.847	.0648*	0.801	n.s.	−0.138	n.s.	**3.153**	.**0016**
Diptera	−0.309	n.s.	0.440	n.s.	−0.469	n.s.	**2.869**	.**0041**	**3.564**	.**0004**
Hemiptera	−0.363	n.s.	0.044	n.s.	1.198	n.s.	−1.894	.0582*	0.041	n.s.
Hymenoptera	0.748	n.s.	−0.555	n.s.	−1.208	n.s.	−0.013	n.s.	−**2.070**	.**0384**
Hymenoptera: Formicidae	**4.853**	**<.0001**	−**5.024**	**<.0001**	−0.935	n.s.	−1.446	n.s.	1.615	n.s.
Lepidoptera	−**2.333**	.**0197**	1.146	n.s.	0.759	n.s.	**4.311**	**<.0001**	0.295	n.s.
Opiliones	−0.148	n.s.	0.210	n.s.	−0.137	n.s.	0.595	n.s.	−0.158	n.s.
Orthoptera	−0.382	n.s.	−0.287	n.s.	1.798	.0721*	0.411	n.s.	**3.220**	.**0013**

Note

Owing to covariance between ground cover categories, listed statistics have resulted from individually assessing each variable separately. Bold text indicates statistical significance at *α* = .05, an asterisk indicates marginal significance at *α* = .10, and “n.s.” indicates lack of significance.

**TABLE 2:** Three most parsimonious models of individual taxonomic group abundance with environmental covariates

Taxon	Covariates	∆ AIC
Acari	**Woody diversity + rock**	0.000
Acari	Woody diversity + rock* **+ **canopy cover	0.995
Acari	**Woody diversity + leaf** litter*	1.083
Araneae	**Insect prey + woody diversity**	0.000
Araneae	Woody diversity	2.041
Araneae	Insect prey	2.092
Blattodea	Woody diversity + vegetation*	0.000
Blattodea	**Woody diversity**	0.804
Blattodea	**Woody diversity + canopy** cover	1.350
Coleoptera	**Woody diversity + canopy cover**	0.000
Coleoptera	**Woody diversity + canopy cover + vegetation**	1.284
Coleoptera	**Woody diversity + canopy cover + rock**	1.588
Collembola	**Woody diversity + rock**	0.000
Collembola	**Woody diversity + leaf litter**	0.858
Collembola	**Woody diversity + rock + **canopy cover	1.900
Diptera	**Woody diversity + canopy** cover*	0.000
Diptera	**Woody diversity + vegetation** cover	1.385
Diptera	**Woody diversity + canopy** cover **+ vegetation**	1.405
Hemiptera	Canopy cover*	0.000
Hemiptera	**Canopy cover + rock**	0.696
Hemiptera	Canopy cover* **+ **vegetation	0.745
Hymenoptera	**Woody diversity**	0.000
Hymenoptera	**Woody diversity + rock**	0.289
Hymenoptera	**Woody diversity + leaf** litter	0.919
Hymenoptera: Formicidae	**Rock + woody diversity + canopy** cover	0.000
Hymenoptera: Formicidae	**Rock + woody** diversity*	0.101
Hymenoptera: Formicidae	**Rock**	0.542
Lepidoptera	**Canopy cover**	0.000
Lepidoptera	**Canopy cover + rock**	0.289
Lepidoptera	**Canopy cover + leaf** litter	0.919
Opiliones	Canopy cover	0.000
Opiliones	All plant diversity	0.096
Opiliones	Leaf litter	0.123
Orthoptera	Vegetation **+ all** plant diversity	0.000
Orthoptera	Vegetation*	0.513
Orthoptera	Woody diversity*	1.591

Bold text indicates statistical significance at *α* = .05, an asterisk indicates marginal significance at *α* = .10, and plain text indicates lack of significance.

**FIGURE 5:**
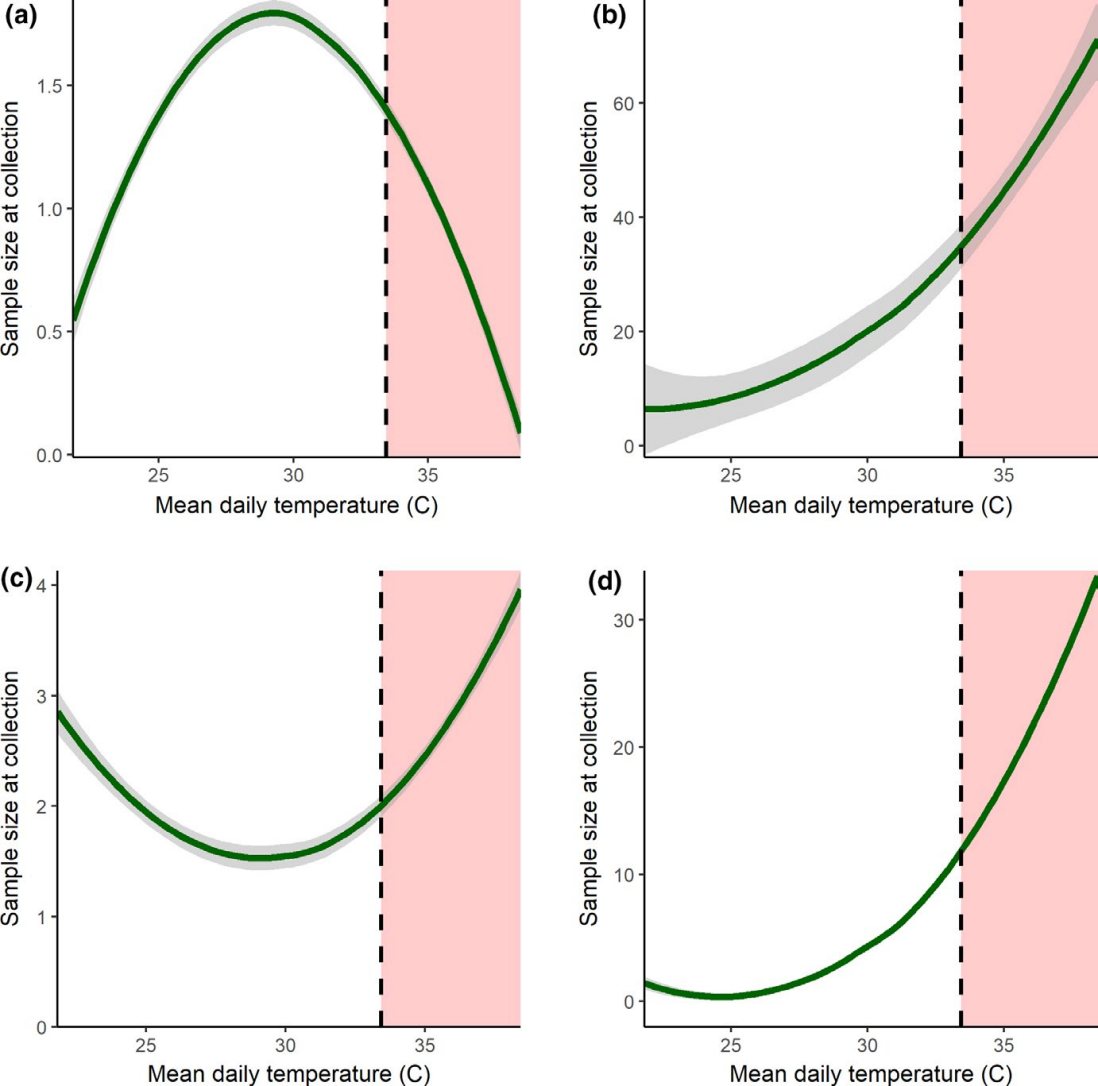
Predicted abundance on sample collection dates of (a) adult spiders (Araneae), (b) ants (Hymenoptera: Formicidae), (c) harvestmen (Opiliones), and (d) crickets (Orthoptera: Gryllidae and Rhaphidophoridae) within observed temperature range. Red shading indicates projected temperature increase by late century (2071–2100), predicted by Gonzalez et al. (2018) for the study area using ensemble means of global circulation models under emissions scenario RCP 8.5

Most arthropod groups were collected too infrequently for separate longitudinal analysis. Cumulative and mean precipitation were less significant in predicting temporal abundance; however, cumulative precipitation was positively correlated with ants (*t* = 2.116; *p* = .035) and positively but marginally correlated with flies (*t* = 1.782, *p* = .076). The temporal abundance of collected spiders was strongly correlated with temperature (*t* = 3.390; *p *< .001) and a polynomial temperature variable (*t* = −3.302; *p* = .001; Figure 5a), but not with cumulative precipitation (*t* = −1.485; *p* = .139). Temperature was also a significant predictor of the abundance of flies, ants, harvestmen, and orthopteran crickets. Although the collected spiders declined to nearly zero at the highest predicted temperatures, flies, springtails, harvestmen, crickets, and ants all steeply increased in abundance under the RCP 8.5 scenario (Figure 5).

## 4 DISCUSSION

The shale barrens of the central Appalachian mountains comprise unique plant communities with significant environmental and physiological stressors imposed upon resident biota. Through this work, we identified the importance of dominant vegetation and other ground cover to broad, epigeic arthropod groups within these habitats. Immediate and near‐term threats to the unique composition of the shale barrens have the capacity to drastically alter arthropod community dynamics and microhabitat selection. Changing climate, forest succession, and resulting mesophication can facilitate understory plant colonization, increase shading, and facilitate further invasion of exotic plants (Nowacki & Abrams, 2008; Tyndall, 2015; West et al., 2009), all of which can greatly affect arthropod communities.

Any changes to the understory and low‐growing vegetation in the barrens resulting from these stressors and threats have the capacity to alter epigeic arthropod community composition. As we had hypothesized, we found more salticid jumping spiders that typically hunt on plant surfaces and fewer ground‐dwelling Gnaphosidae in traps surrounded by greater vegetation cover. This vegetation cover provides an enhanced landscape upon which salticid spiders are able to hunt while limiting open ground for gnaphosid spiders that exploit prey on the ground surface. While our pitfall traps mostly captured cursorial taxa, with increased vegetation cover we would expect to see community shifts toward web‐building spiders and those that hunt on vegetative surfaces and away from the ground‐active taxa (Hurd & Fagan, 1992). For these groups, vegetative structure may be a limiting resource in the barrens and other habitats with sparse vegetation ground cover; thus, changes in plant community composition could result in a dramatic shift of the dominant spider taxa (Landsman & Bowman, 2017; Takada et al., 2008). Shifts in the insect community are also likely to occur, particularly as the extent of herbaceous and woody vegetation increases and subsequently increases available food and oviposition sites for diverse herbivorous insect families in the Hemiptera and Coleoptera. However, changes to the extent of shaded microhabitats and low plant ground cover may also reduce habitat for adapted barren species and specialist pollinators (Kalhorn et al., 2003).

Of more ecological importance to the arthropod community than the herbaceous ground cover was the role of overstory trees which provided shaded microhabitats and leaf litter on the ground surface. We found a marginally significant correlation between accumulated leaf litter and detritivorous springtails, likely owing to their diet (Triplehorn & Johnson, 2005). Springtails are an important prey source for wolf and other hunting spiders, so a reduction in springtail abundance in areas with less leaf litter could consequently reduce spider habitat use (Foelix, 2011). We also noted an increase in beetles and ants in plots with reduced leaf litter and increased bare rock cover. Anecdotally, carabid and tenebrionid ground beetles were particularly common in our traps, with many being adapted to high ground temperatures (May, 1979; Ward & Seely, 1996). Some ant groups are similarly resilient and are able to evolve and adapt to hot microhabitats that may not be suitable for other taxa (Angilletta et al., 2007; Diamond et al., 2017). The extent of canopy cover was also correlated with numerous groups, including beetles, mites, flies, cockroaches, and moths. Particularly when considering the necessary thermoregulatory behaviors of many ectotherms, the extent and availability of shaded microhabitats is extremely important for lowering body temperatures in high ambient temperatures (Kearney et al., 2009). Additionally, the species diversity of woody shrubs and trees was one of our most significant covariates, affecting the abundance of most analyzed invertebrate taxa. The significance of this relationship may result from both herbivore‐plant associations and the likely increase in habitat structural complexity associated with a more diverse woody plant community. The relationship between trees and arthropod groups in shale barren habitats, mediated through increased abundance of leaf litter and shaded microhabitats, illustrates the influence that the extent of overstory vegetation holds over shale barren habitats and their inhabitants.

Beyond site‐specific habitat traits, climatic factors, particularly air temperature, are also significant in determining relative abundance and community structure. Diel and seasonal temporal patterns of many arthropod groups are strongly correlated with temperature. Invertebrate physiological development throughout the growing season, particularly for univoltine taxa, are strongly correlated with air temperature and photoperiod, which collectively drive the timing of important life history events such as instigation of diapause, oviposition, and molting. This could be reflected in the polynomial response of spider capture to temperature gradients that we noted in our study. Depending on the specific spider species captured, this polynomial relationship could be caused by the phenological development of univoltine taxa: as adults produce young and die, the number of captured adults decrease with subsequent increases in the late season as the young develop. As poikilothermic organisms, daily surface activity is nearly wholly dependent upon air temperatures, with organisms seeking shade or shelter when local climate exceeds optimal air temperature (Humphreys, 1987; Karasov & Martínez del Rio, 2007). This could also be reflected in the correlation between spider capture and temperature as surface activity was highest at optimal air temperatures but suppressed at higher temperatures. At these higher temperatures, spiders may be seeking out and sheltering in shaded microhabitats to avoid direct irradiance and cool body temperatures (Foelix, 2011; Humphreys, 1987; Pulz, 1987). The reduction in spider captures at higher temperatures could also be indicative of heat‐induced mortality of some individuals. The mean daily maximum air temperature values for collection periods reached a high of 33.43°C with recorded daily maximum high temperature of 37.78°C. Throughout the field season, daily temperatures exceeded 33°C on 33 days or for nearly 22% of the sample period. Thermal‐induced mortality has been recorded for wolf spiders at or near 40°C, with thresholds for other ground‐dwelling spiders ranging between 37 and 46°C (Almquist, 1970; Davis, 1989). Although we did not directly measure temperatures at the ground surface in this study, surface temperatures are known to exceed 60°C in shale barrens (Keener, 1983).

In contrast to the pattern noted with spiders, both ants and crickets continued to be found in greater abundance at the higher temperatures in the sample season. Orthopterans may be more resilient to and active in increased temperatures (Gilman et al., 2008), and experimental evidence has shown developmental and immunological benefits at temperatures approximate to the noted mean daily maximum during our sample season (Adamo & Lovett, 2011; Sangbaramou et al., 2018). Ants exhibit certain behaviors that may prevent negative effects of elevated daily temperatures, including an ability to move larvae and eggs deeper into subsurface soils and a partly fossorial life history (Jones & Oldroyd, 2006; Seeley & Heinrich, 1981). However, the use of such thermoregulatory behaviors is limited by energetic requirements and time needed for foraging or hunting (Kearney et al., 2009). The projected response to increased temperatures is built from observational data and as such, does not reveal any physiological thresholds for these taxa. At these elevated temperatures, there would certainly be a point at which air temperatures begin to negatively affect these groups, despite increases in their abundance within the realized climatic environment.

As evidenced herein and in numerous other subsequently listed works, air temperature plays a critical and influential role in various aspects of invertebrate life history. Elevated air surface temperatures from climate change could affect life history traits such as fecundity and reproductive capacity (Chen et al., 2010; Grazer & Martin, 2012); alter surface activity and copulatory behavior (Jiao et al., 2009; Katsuki & Miyatake, 2009; Pulz, 1987); affect organismal development (Abril et al., 2010; Atkinson, 1994; Kiss & Samu, 2002); reduce survival of individuals at varying life stages (Abril et al., 2010; Almquist, 1970; Davis, 1989); and shift phenological relationships between producers and consumers (Menzel et al., 2006). Within the study area, Gonzalez et al. (2018) projected that annual average air temperatures will increase by 5.0 ± 1.0°C from 1971–2000 to 2071–2100, with precipitation expected to increase by 14.0%, mostly in the form of winter precipitation (Gonzalez et al., 2018; Wuebbles et al., 2017). These projections, however, are for air temperatures averaged throughout the year and do not consider extreme temperatures in the summer or throughout the growing season only. Additionally, projected temperatures do not account for ground surface temperatures which can greatly exceed that of air temperatures in these habitats (Keener, 1983). Projected changes to the local climate, including longer growing seasons and warmer daily temperatures, are likely to affect arthropod community structure by altering relative taxon abundances and subsequently influencing biotic interactions between species and trophic groups (Ockendon et al., 2014). While warmer temperatures may further reduce the extent of competing vegetation, longer growing seasons may facilitate plant growth and forest succession in marginal habitats that contain more leaf litter and herbaceous species (Keenan et al., 2014). A longer growing season may also allow for certain organisms to produce additional clutches, changing to a multivoltine reproductive strategy (Altermatt, 2010; Gomi et al., 2007; Jönsson et al., 2009). For species that overwinter as juveniles, longer growing seasons may result in early maturation with unknown consequences to overwintering survival (Kiss & Samu, 2002). Epigeic arthropods must also adapt to secondary effects of climate change mediated through changes in vegetation and canopy cover, both factors that were significant predictors of various taxonomic groups within the study area. Shifts in arthropod community structure resulting from changes in precipitation, albeit less studied, may also result from projected climatic changes, with differential impacts to various functional groups (Zhu et al., 2014).

While we were able to detect significant and marginally significant correlations between arthropod taxonomic orders and our covariates, we likely failed to detect other relationships that would be identified with finer taxonomic resolution (Mueller et al., 2013; Terlizzi et al., 2009). Greater accuracy in identifications may illustrate similar but stronger correlations (Landsman et al., 2021; Timms et al., 2013); however, the low abundance of many collected taxa may preclude the ability to detect species‐specific correlations upon appropriate error adjustment. Using coarse taxonomic generalizations herein met our objectives by providing preliminary investigation into the arthropod communities of the unique shale barren habitats (Meehan et al., 2019). Future work in similar habitats should include finer resolution of identified taxa to further elucidate correlations of specific families, genera, or species. Owing to the sensitivity of the barrens to physical disturbances, this work focused on a single site representative of the shale barren habitats to limit any potential adverse impacts from sampling. Repeated visits to the study site to maintain traps notably facilitated minor surficial shale erosion: future work in these habitats should balance the importance of greater sample sizes with the physical sensitivity of the barrens.

Shale barrens, in addition to other unique, insular habitats, may experience disproportionate negative impacts from changing climates and subsequent shifts in dominant vegetation (Cartwright, 2019). Conversely, such insular habitats may also provide diverse and heterogeneous microhabitats and thus function as more permanent refugia for certain taxa, due in part to the unique physiognomy of the landscape (Speziale & Ezcurra, 2014). As described by Cartwright (2019), insular habitats are often physiologically stressful environments, which beget the endemism and adaptations seen in shale barren plant and animal species. Under projected elevated air surface temperatures, biological stress on heliophytic native shale barren species from competition with other plants could be reduced; however, certain native plants and arthropods may become overly physiologically stressed, already existing in an environment that experiences extreme ground surface temperatures. The extent of overstory trees and associated shading may provide temporary respite from extreme temperatures for many arthropod taxa but may conversely be detrimental to the endemic plant species and heat‐resilient insects adapted to the barren habitats. Changes to this plant group could consequently result in further impacts to endemic plant species, the availability of shaded microhabitats, and adapted arthropod communities. Exotic species invasion, potential changes to vegetation cover, and inhospitable matrix habitats between insular barren patches thus create a multifaceted conservation concern for State and Federal public land managers (Frye & Neel, 2016). Coupled with rising future temperatures and subsequent shifts in the extent of ground and overstory vegetation, successful conservation of shale barren habitats throughout their range must not only focus on current threats, but the condition of projected future environments as well. This undertaking provides a critical, preliminary investigation into the epigeic arthropod communities of the shale barrens and their correlations with local‐scale environmental conditions; however, further work is needed to fully understand these unique habitats and to understand how environmental changes may influence arthropod communities.

## CONFLICT OF INTEREST

None declared.

## AUTHOR CONTRIBUTIONS

**Andrew P. Landsman:** Conceptualization (lead); Formal analysis (lead); Writing – original draft (lead). **Clara R. Thiel:** Writing – original draft (supporting); Writing – review & editing (supporting).

## Data Availability

Data are available from the United States National Park Service data repository at https://irma.nps.gov/DataStore/Reference/Profile/2288289.
